# P2X7 receptor-mediated TG2 externalization: a link to inflammatory arthritis?

**DOI:** 10.1007/s00726-016-2319-8

**Published:** 2016-08-25

**Authors:** Daniel Aeschlimann, Vera Knäuper

**Affiliations:** 1 Matrix Biology and Tissue Repair Research Unit, School of Dentistry, College of Biomedical and Life Sciences, Cardiff University, Heath Park, Cardiff, CF14 4XY UK; 2 Arthritis Research UK Biomechanics and Bioengineering Centre of Excellence, College of Biomedical and Life Sciences, Cardiff University, The Sir Martin Evans Building, Museum Avenue, Cardiff, CF10 3AX UK

**Keywords:** Transglutaminase, P2X7 receptor, Purinergic signaling, Arthritis, Cartilage, Inflammation, Autoimmunity

## Abstract

Transglutaminases have important roles in stabilizing extracellular protein assemblies in tissue repair processes but some reaction products can stimulate immune activation, leading to chronic inflammatory conditions or autoimmunity. Exacerbated disease in models of inflammatory arthritis has been ascribed to sustained extracellular enzyme activity alongside formation of select protein modifications. Here, we review the evidence, with a focus on the link between P2X7R signaling and TG2 export, a pathway that we have recently discovered which ties extracellular protein modifications into the danger signal-mediated innate immune response. These recent insights offer new opportunities for therapeutic intervention.

## Introduction

A role of transglutaminases (TG) in formation of skeletal tissues was postulated based on functional in vitro studies and by linking the expression of several of the enzymes belonging to this protein family to the developmental program (Aeschlimann and Thomazy 2000). Recent experimental evidence in support of the role of TGs in cartilage development and homeostasis is eloquently outlined in a review by Adamczyk in an accompanying article in this issue of Amino Acids (Adamczyk 2016). While TG2−/− mice had no overt developmental abnormalities (Nanda et al. 2001; De Laurenzi and Melino 2001), deficiencies became apparent once the mice were subjected to injury or challenged in experimental models of disease (Iismaa et al. 2009). This highlighted two points: First, that TG2 is dispensable for skeletal development, a fact that is further reinforced by the absence of overt skeletal abnormalities and grossly normal bone mineral content in TG2 and factor XIIIa double knock-out mice (Cordell et al. 2015). Second, that the inflammatory response in TG2−/− mice is substantially altered, which often results in delayed or compromised tissue repair but may also offer protection in certain circumstances, for example following CNS injury or in neurodegenerative conditions. A detailed discussion of this is beyond the scope of this review, and we will focus here on aspects relevant to joint disease.

## TG2 externalization occurs in the context of inflammation

Although having well characterized extracellular functions, TG2 is externalized by cells through an unconventional secretion pathway (Aeschlimann and Paulsson 1994), the details of which remain to be completely deciphered. We recently identified that P2X7 receptor (P2X7R) activation controls active TG2 secretion by cells (Adamczyk et al. 2015). This not only established for the first time a model in which the steps leading to TG2 externalization can now be meaningfully interrogated (P2X7R expressing HEK293 cells) but also, importantly, provided a mechanistic explanation for a link between extracellular TG2 activities and inflammation. P2X7R is a member of the P2X family of nucleotide gated ion channels that is activated by high concentrations of extracellular ATP (Hattori and Gouaux 2012). Ion channel opening allows K^+^ efflux that triggers inflammasome assembly in innate immune cells in a NLRP3-dependent manner (Strowig et al. 2012), leading to caspase-1 autoprocessing and ultimately, maturation and secretion of proinflammatory interleukin (IL)-1 family cytokines (Mariathasan et al. 2006). Given its requirement for high extracellular ATP concentration, P2X7R will primarily be activated following injury or in the context of inflammation, where P2X7R-mediated release of ATP from immune cells acts as a danger signal amplification system. TG2 expression is highly upregulated by acute phase injury cytokines (Aeschlimann and Thomazy 2000; Nurminskaya and Belkin 2012), and hence abundant P2X7R-driven TG2 release will occur in such an environment. Furthermore, co-secretion of thioredoxin (Adamczyk et al. 2015), an oxidoreductase enzyme, will prevent inactivation of TG2 which would readily occur in the oxidative inflammatory environment through the redox sensitive Cys switch mechanism of TG2 (Stamnaes et al. 2010a; Jin et al. 2011). In combination, this will promote the accumulation of sustained high levels of active extracellular enzyme. The role of TG2 in promoting TGF-β activation (Kojima et al. 1993; Szondy et al. 2003) may, hence, have a critical function in terminating the proinflammatory cascade as TGF-β signaling can render cells inert to proinflammatory signals (Koutoulaki et al. 2010), facilitating resolution of the inflammatory response.

IL-1β family cytokines and thioredoxin-1 similar to TG2 are leaderless proteins that are not targeted to the classical ER to Golgi pathway for export (Rubartelli et al. 1990, 1992), and their precise mechanism of secretion remains a matter of debate. It is possible that all or part of the mechanism guiding the release of these three proteins is shared, particularly as we have shown that TG2 and thioredoxin are co-secreted (Adamczyk et al. 2015). A common mechanism that enables rapid deployment of these proteins is also consistent with their overlapping functions in innate immunity. Several distinct mechanisms that can support unconventional protein secretion have been identified (for review see Nickel and Rabouille 2009; Rabouille et al. 2012), and microvesicle shedding at the plasma membrane, exocytosis of endo-lysosome-derived vesicles or transporter-facilitated direct membrane translocation implicated in IL-1β secretion (Eder 2009). We have been able to mechanistically separate P2X7R-stimulated vesicle release from TG2 export, and have shown that TG2 is directly secreted across the plasma membrane in free form (Adamczyk et al. 2015). Our data also suggest that TG2 secretion is independent of inflammasome assembly but instead relates to the ability of P2X7R to induce ‘membrane pores’ (Adamczyk et al. 2015; discussed below). Interestingly, recent data show that IL-1β secretion can also be de-coupled from NLRP3 or AIM2 inflammasome formation and its maturation by caspase-1 processing, and is mediated by a state of membrane hyperpermeability (Martín-Sánchez et al. 2016). Although single cell analysis supports bulk release of IL-1β in the context of inflammasome activation-driven cell death (Shirasaki et al. 2014), inflammasome-independent P2X7R-driven IL-1β secretion has been demonstrated in cell models (Gudipaty et al. 2003), may have distinct biological functions, notably in non-immune cells, and may relate to P2X7R activation-mediated membrane pore formation that is reversible. Nevertheless, activation of caspase-4/5 can trigger a form of programmed cell death termed pyroptosis. Pyroptosis is part of the innate immune defense to infection and features plasma membrane pore formation that ultimately results in fragmentation of infected cells. Gasdermin D was recently shown to be a critical effector component of the canonical NLRP3, AIM2, and NAIP-NLRC4 inflammasome pathways, substantially impacting on IL-1β secretion without affecting caspase-1 autoprocessing or IL-1β activation (Shi et al. 2015; Kayagaki et al. 2015). Cleavage of gasdermin D by inflammatory caspases-4/5 or -1 leads to dissociation of gasdermin N-domain from its autoinhibitory C-domain and results in formation of large membrane pores (Aglietti et al. 2016; Ding et al. 2016). Whether gasdermin D N-domain pores also support release of TG2 and thioredoxin from cells undergoing pyroptosis remains to be investigated.

## Role of the P2X7R-TG2 pathway in rheumatoid arthritis

Rheumatoid arthritis (RA) is a chronic autoimmune disease characterized by specific adaptive immune cell responses, synovial hyperplasia and inflammation-driven cartilage and bone destruction. Citrullination of proteins by members of the peptidyl arginine deiminase (PAD) family of enzymes (primarily PAD4 but PAD2 and PAD3 are also involved) is a characteristic feature of disease (Harris et al. 2008), and the resulting neo-epitopes elicit an immune response via a mechanism that shares some similarity to the pathogenesis of celiac disease (Molberg and Sollid 2006). Antibodies to citrullinated peptides (anti-CCP antibodies or ACPA) signify disease development, and have become an accepted marker in diagnosis (Liao et al. 2013). More recently, a pathogenic loop involving PAD3/PAD4-reactive autoantibodies that activate PAD4 and thereby drive the formation of immune-stimulating epitopes has been implicated in rapid disease progression (Darrah et al. 2013). Current therapeutic approaches target aspects of immunity (blocking TNF-α or targeting B cells) but a substantial fraction of patients are nonresponsive to these treatments, highlighting first, the fact that pathogenesis is not uniform and second, the need to identify the implied additional pathways that drive joint destruction.

**Fig. 1:**
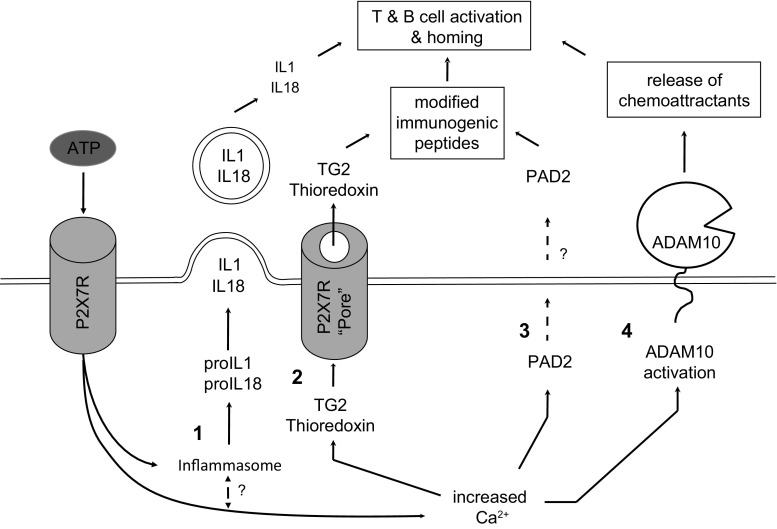
Schematic outlining purinergic signaling-mediated events contributing to inflammatory joint destruction. *1* Inflammasome assembly is initiated following Toll-like receptor engagement (signal 1, not shown) and intracellular K^+^ depletion in response to P2X7R channel opening (signal 2), leading to caspase-1 mediated processing of pro-IL-1β/pro-IL-18 into the proinflammatory mature cytokines (Strowig et al. 2012). Subsequent release of cytokines can occur via microvesicle shedding at the plasma membrane (induced by P2X7R via MAPK p38 and Rho pathways), although several alternative mechanisms have been proposed (Eder 2009; Martín-Sánchez et al. 2016). Recent evidence suggests that ‘bulk’ release of IL-1β may be largely a consequence of pyroptosis, a form of cell necrosis that is triggered by formation of large gasdermin D membrane pores upon inflammasome activation (Shirasaki et al. 2014; Shi et al. 2015). *2* TG2 secretion in response to P2X7R activation depends on the membrane pore functionality of the receptor (Adamczyk et al. 2015). However, it appears to be independent of inflammasome activation, given that P2X7R-mediated TG2 secretion can be transferred to a HEK cell model that lacks inflammasome components and secretion is unaffected by caspase-1 inhibition in macrophages (Adamczyk et al. 2015). Thioredoxin, an activator of TG2, is co-secreted with TG2 (Adamczyk et al. 2015), and consequential thioredoxin depletion from thioredoxin-interacting protein (TXNIP) intracellularly was shown to induce inflammasome assembly and drive the release of thromboinflammatory particles by macrophages (Rothmeier et al. 2015). *3* PAD2/4 release and activation is induced in neutrophil extracellular traps (NETs) leading to extensive citrullination of extracellular proteins in RA (Spengler et al. 2015). This implicates inflammatory cell death in this process, consistent with its induction in experimental models by the phorbolester PMA or the calcium ionophore ionomycin (Blachère et al. 2015). However, citrullination of proteins during NETosis in the joint is unlikely to represent the event leading to the original breakdown of immune-tolerance to citrullinated peptides. A rise in intracellular Ca^2+^ levels in response to P2X7R activation in mast cells was recently shown to lead to PAD2 release and activation in the absence of cell death (Arandjelovic et al. 2012), suggesting that the danger signal ATP may drive this process in the initial phase during disease development. *4* P2X7R signaling mediates activation of ADAM-10 via intracellular signaling, and this is likely due to the rise in intracellular Ca^2+^ that follows P2X7R channel opening (Horiuchi et al. 2007). ADAM-10 activation results in shedding of a variety of cell surface proteins that have key regulatory roles in inflammation, for example lymphocyte trafficking via IL-6 *trans*-signaling (Garbers et al. 2011)

Activation of P2X7R may drive accumulation of extracellular TG, and ultimately TG-mediated protein modification or crosslinking reactions that have a role in pathogenesis. In support of this, mouse models of disease linked both TG2 and factor XIIIa reaction products mechanistically to an exacerbated inflammatory response that drives disease progression and joint destruction (Dzhambazov et al. 2009; Raghu et al. 2015). Given the role of the NLRP3-dependent inflammasome pathway in proinflammatory cytokine production, unsurprisingly P2X7R−/− mice were protected from inflammatory arthritis as shown using the collagen type II (CIA)-induced arthritis model (Labasi et al. 2002). P2X7R−/− mice also do not develop Freund’s adjuvant (CFA)-induced chronic inflammatory hypersensitivity (Chessell et al. 2005). However, it is noteworthy that TG2 has been shown to modify epitopes targeted by T cells in the CIA model, and to exacerbate incidence, severity, and histopathological features of disease (Dzhambazov et al. 2009). Notably, injection of functional but not inactive enzyme also triggers a B cell response to the enzyme itself, an event that may originate from complex formation of the enzyme with peptides containing T cell epitopes in a process akin of what is seen in celiac disease (Stamnaes et al. 2010b). Interestingly, it has also been shown that P2X7R activation in mice drives PAD2-mediated protein citrullination, an event linked to anti-CCP antibody development in RA (Arandjelovic et al. 2012). Hence, ablation of P2X7R may have effects on the immune response that go beyond suppressing formation of biologically active IL-1 and IL-18, namely, also suppressing the formation of posttranslational protein modifications that are targeted by the adaptive immune system (Fig. 1).

The efficacy of P2X7R antagonists has been extensively examined in rodent models of inflammatory arthritis, with some success (for summary see Table 7 in Bartlett et al. 2014; McInnes et al. 2014). Blocking P2X7R suppresses synovial inflammation substantially and reduces local tissue damage as well as mechanical hyperalgesia, particularly when administered prior to disease onset, with no apparent effect on the systemic acute phase response. Confirmatory clinical studies are underway but have so far not shown the expected efficacy (Keystone et al. 2012; Stock et al. 2012). One reason for this could be the highly polymorphic nature of the *P2RX7* gene in the human population. It is becoming increasingly clear that a growing number of amino acid substitutions found in P2X7R have a substantial impact on receptor functionality (Stokes et al. 2010), and some strongly predispose to chronic inflammatory diseases, whereas others offer protection. Indeed, SNP linkage analysis in an RA cohort revealed a positive correlation with the presence of a gain-of-function P2X7R allele (Al-Shukaili et al. 2011) which we have shown to mediate enhanced TG2 release (Adamczyk et al. 2015). Hence, it may be necessary to consider the *P2RX7* genotype when evaluating the efficacy of P2X7R antagonists, as antagonist binding affinity or baseline receptor activation state are P2X7R variant-specific and can differ substantially. Indeed, receptor variant-dependent pharmacodynamics has been reported for one of the antagonists in development (McHugh et al. 2012).

The mechanism by which TG2 contributes to RA progression is not completely understood. TG2 is overexpressed in human RA lesions (Weinberg et al. 1991), and the presence of active TG2 substantially increases severity of disease in the CIA model (Dzhambazov et al. 2009) whereas a virally transduced localized knockdown of TG2 appears to alleviate joint destruction (Lauzier et al. 2012). As administration of TG2 alone in the absence of collagen II immunization does not elicit an immune response, and as functional enzyme but not inactive TG2 exacerbates the disease course, this suggests that TG2 does not initiate the autoimmune response but that TG2-catalyzed reactions modify the immune response (Dzhambazov et al. 2009). The fact that the increased disease severity is not localized to the immunization site but systemically affects joints further suggests that the altered disease course is a consequence of exacerbated adaptive immunity (Dzhambazov et al. 2009), and this likely involves targeting of neo-epitopes generated by TG2. However, although Q^267^ in the immunodominant collagen II T cell epitope (IAGFKGEQGPK) can be deamidated by TG2, this does not lead to enhanced presentation or T cell stimulation (Dzhambazov et al. 2009). It is possible that other, as yet unidentified epitopes targeted by T cells are generated by TG2. Alternatively, the explanation could also be the development of a B cell response to TG2. With circulating autoantibodies, immune-complex formation at RA lesion sites is likely to occur and promoted by inflammation-driven TG2 overexpression and externalization, and hence could contribute to exacerbated disease. Indeed, a B cell response to TG2 is seen only following administration of functional enzyme (Dzhambazov et al. 2009), and anti-TG autoantibody-driven pathogenesis has been implicated in extraintestinal manifestations of celiac disease (Boscolo et al. 2010; Zone et al. 2011). However, while anti-TG2 antibodies have been reported in RA patients and other immune-mediated forms of arthritis in some studies (Picarelli et al. 2003), it is not a prevalent or consistent feature of human RA (Liao et al. 2013).

In contrast to TG2, factor XIIIa does not apparently alter T and B cell responses in the CIA model but plays a role in differentiation of myeloid precursor cells into their mature progenies including osteoclasts (Raghu et al. 2015). Nevertheless, factor XIIIa−/− mice display an attenuated proinflammatory response. It remains to be investigated whether this relates to crosstalk between the immune system and the coagulation cascade, leading to enhanced plasma factor XIII zymogen activation and fibrinogen deposition which drives inflammation. Alternatively, this may relate to externalization of the catalytic subunit (a_2_-form) by myeloid cells which could have direct, coagulation system-independent functions.

## Role of P2X7R and TG2 in inflammation associated with gout

Enhanced TG2 expression by synovial mononuclear cells from patients with gouty arthritis is associated with increased production of bioactive TGF-β (Yen et al. 2015). TG2 has also been implicated in the clearance of apoptotic cells by phagocytes in acute inflammation models (Szondy et al. 2003), including a mouse model of gout-like inflammation where it is thought to facilitate clearance of apoptotic neutrophils by macrophages (Rose et al. 2006). The mechanism for this appears to involve interactions of extracellular TG2 with β3-integrin and MFG-E8 but is independent of catalytic activity (Rose et al. 2006; Tóth et al. 2009). TG2 secretion normally brings about its activation through Ca^2+^-induced conformational changes (Pinkas et al. 2007). However, it is possible that the high concentrations of extracellular nucleotides present at sites of inflammation or an interaction with heparan sulfate-bearing cell surface proteins (Lortat-Jacob et al. 2012) stabilizes the nucleotide-bound conformation and thereby prevents Ca^2+^-binding and activation. The importance of TG2 in regulating inflammation in this context was further substantiated by the fact that TG2−/− mice exhibited an exacerbated inflammatory response in the acute gout-like peritoneal inflammation model (Yen et al. 2015). Hyperuricemia and gout are metabolic diseases caused by purine metabolism disorder. Gout has many manifestations including chronic inflammatory arthritis, treatment of which remains a challenge. Mechanistically, hyperuricemia, i.e., uric acid, the end product of purine metabolism, drives monosodium urate crystal (MSU) formation (Martillo et al. 2014). MSU crystals activate the immune system via toll-like receptor activation and inflammasome signaling. An acute episode may be brought about by stimulation of synovial macrophages and monocytes to release large amounts of proinflammatory IL-1β and IL-18 (Rock et al. 2013), a view that is supported by IL-1 antagonism providing clinical benefit in patients with gout-associated arthritis (Schlesinger et al. 2012). Epidemiological studies have shown that only about 10 % of patients (range 2–36 % depending on study) with hyperuricemia will develop gout suggesting that other factors play an overriding role. While genetic variations in P2X7R are suspected of contributing to disease (Gong and Chen 2015), no such link has been made for TG2. Attention has switched to immune activation as a cause because immunoglobulins from the synovial fluid of patients with gout but not other forms of arthritis promote MSU crystal formation. Decoration of crystals with immunoglobulins drives inflammation through activation of Fc-receptor bearing cells. Interestingly, MSU immunized B cell-deficient mice displayed reduced effector T cell function, and uric acid-induced immune activation could be restored by antibody transfer supporting that MSU crystals evoke a danger signal response (Kanevets et al. 2009).

## P2X7R-TG2 pathway in osteoarthritis: a link to inflammation-driven pain?

P2X7R expression is not restricted to the hematopoietic lineage but it is widely expressed in many tissues (Bartlett et al. 2014) including the musculoskeletal system where ATP release in response to mechanical loading has been postulated to have a regulatory role in tissue homeostasis (Garcia and Knight 2010). P2X7R is expressed by chondrocytes and, hence, exposure of cells to excessive mechanical stress in osteoarthritis (OA) may lead to ATP release, which in turn may trigger TG2 secretion through activation of P2X7R. Hence, both tissue intrinsic TG2 released by chondrocytes themselves as well as associated with the inflammatory response could contribute to the elevated levels of γ-glutamyl-ε-lysine crosslinks present in OA tissue (Huebner et al. 2009).

P2X7R has several activation states; ATP stimulation initially causes ion channel opening, that besides K^+^ efflux supports Ca^2+^ and Na^+^ influx, leading to membrane depolarization and activation of intracellular signaling cascades (Coddou et al. 2011; Bartlett et al. 2014). This is functionally linked to a disintegrin and metalloproteinase (ADAM)-10 activation, which leads to chemoattractant release that supports lymphocyte homing (Fig. 1) (Garbers et al. 2011). Prolonged ATP exposure leads to formation of a ‘membrane pore’ that enables membrane permeability to larger organic cations (Virginio et al. 1999; Browne et al. 2013). The identity of this pore remains controversial as there is conflicting evidence suggesting either dilation of the P2X7R channel itself or an interaction of P2X7R with another plasma membrane channel, potentially identified as pannexin-1. However, recent studies demonstrate that inflammasome activation is pannexin-1 independent (Qu et al. 2011; Fowler et al. 2014). Interestingly, mutations in P2X7R that interfere with membrane pore formation have been associated with reduced chronic pain in OA patients (Sorge et al. 2012). Studies in animal models highlighted the role of microglia cell-produced proinflammatory cytokines in hypersensitivity to pain, and demonstrated that P2X7R pore formation is responsible for neuropathic pain sensing (Sorge et al. 2012; Nieto et al. 2016). As TG2 externalization is also controlled specifically by P2X7R membrane pore activity (Fig. 1) (Adamczyk et al. 2015) and extracellular active TG2 is therefore likely to be present in this context, it would be interesting to test whether it has a role that affects pain signaling.

## Conclusions

Based on our recent work and this literature review, we conclude that there is potential for the pathological role of TG2 contributing to chronic inflammation and autoimmunity to be targeted with P2X7R antagonists. Importantly, P2X7R inhibition blocks acute release of large amounts of soluble TG2 by macrophages but has no apparent effect on the level of cell surface-associated enzyme (Adamczyk et al. 2015) that has a critical function in the phagocytic activity of these cells. Recent evidence suggests that an aspect of P2X7R functionality known as ‘membrane pore formation’ is more important than the ion channel activity of this receptor in inflammation. As TG2 externalization is likewise mediated by the P2X7R membrane pore functionality, selectively targeting this activity of the receptor is likely to be more effective therapeutically and this also reduces the risk of undesired side effects. Development of suitable pharmacological inhibitors is an area currently under intense investigation. Unexpectedly, nucleoside reverse transcriptase inhibitors currently used as anti-viral agents have been shown to selectively block large membrane pore activity (Fowler et al. 2014), and hence, may offer for the first time an opportunity to test the efficacy of selective therapeutic intervention.
